# Sleep Quality in Type 2 Diabetes Mellitus Patients Attending a Tertiary Care Hospital in West Bengal: A Cross-Sectional Study

**DOI:** 10.7759/cureus.46163

**Published:** 2023-09-28

**Authors:** Dr. Richa Richa, Namrata Datta, Vikash Raj, Rakesh Kumar, Vinayagamoorthy Venugopal

**Affiliations:** 1 Community and Family Medicine, All India institute of Medical Sciences, Deoghar, Deoghar, IND; 2 Community Medicine, IQ City Medical College, Durgapur, IND; 3 Orthopaedics, All India Institute of Medical Sciences, Deoghar, Deoghar, IND

**Keywords:** physical quality of sleep index, quality of life, psqi, sleep quality, type 2 diabetes mellitus

## Abstract

Background

Patients with type 2 Diabetes Mellitus (DM) have a poor quality of life because of various clinical effects. The sleep quality among these patients is affected and they encounter challenges to sleep and wakefulness due to physiological imbalance and co-morbid sleep pathologies. The present study was conducted to ascertain the sleep quality in type 2 DM patients attending a tertiary center in West Bengal.

Methodology

It was a hospital-based cross-sectional study that was conducted among old/follow-up patients who were suffering from DM. The eligible subjects were selected by systematic random sampling and were interviewed using a semi-structured and pre-tested questionnaire. Information pertaining to socio-demographic characteristics and sleep quality was ascertained. The sleep quality was assessed by using the Pittsburgh Sleep Quality Index (PSQI). Data was analyzed using Statistical Package for Social Sciences (SPSS) version 20 and statistical association of different parameters was tested using Chi-Square test.

Results

A total of 192 subjects were selected for study. Maximum subjects were above 50 years. A total of 102 males and 90 females participated in the study. A total of 110 subjects were poor sleepers (i.e. PSQI score ≥5) while 82 were good sleepers(PSQI<5). Poor sleepers were maximum in the 20-35 years age group, more in females and unemployed individuals. Subjects with poor diabetes control, i.e. HbA1C more than 7gm/dl were poor sleepers.

Conclusions

A higher proportion of patients with type 2 DM had poor quality of sleep. It is important on the part of the health personnel to attend to the sleep issues and impaired quality of life due to sleep inadequacy in DM patients.

## Introduction

Diabetes mellitus (DM) is one of the most chronic medical conditions confronted in clinical practice. Diabetes mellitus has reached epidemic proportions worldwide. The World Health Organization (WHO) has commented, " there is an apparent epidemic of diabetes which is strongly related to lifestyle and economic change”. Globally there were around 415 million people with diabetes in the year 2015; this figure is predicted to rise to 642 million by the year 2040 [1].

The number of people with type 2 diabetes is increasing in every country, and more than 80% of them live in low and middle-income countries such as India, Bangladesh, Bhutan, Pakistan, Sri Lanka, Philippines and Indonesia. India ranks second among the top 10 countries in terms of number of people having diabetes. Presently, 69.2 million have diabetes in India and another 36.5 million people are diagnosed with pre-diabetes, which is a high-risk condition for diabetes and cardiovascular diseases [1,2].

Patients with DM, have a poor quality of life because of its various clinical effects. The sleep quality among these patients is remarkably marred. Diabetes mellitus patients encounter challenges to their sleep and wakefulness due to physiological imbalance and co-morbid sleep pathologies. Clinical research has shown that up to one-third of patients with DM suffered from concomitant sleep disorders, as compared with 8.2% of controls without DM 2. In yet another study more than half of the patients with type 2 DM have been reported as “poor sleepers”[3,4]. Studies have also reported that the high prevalence of poor sleep quality among people with type 2 DM has a negative impact on glycemic control [5,6].

In India, diabetes is one of the major health concerns with an increasing prevalence due to rapid urbanization and changing lifestyles. Few studies have been conducted in India on the quality of sleep among patients with diabetes mellitus and in the eastern part of India, hardly any studies were conducted. Therefore, the current study was undertaken in the Integrated Diabetes and Gestational Diabetes Clinic (IDGDC) of IQ City Medical College and NM Hospital to ascertain the sleep quality in type 2 DM patients attending this clinic and to determine the factors associated with sleep quality.

## Materials and methods

Study design and participants 

This was a hospital-based cross-sectional study that was conducted over a period of two months (January to February 2020) at the Integrated Diabetes and Gestational Diabetes Clinic (IDGDC) of IQ City Medical College, Durgapur. The study was approved by the Institute Ethics and Research Committee before its implementation. All old and follow-up patients giving their consent, between the age 20-65 years, and having type 2 diabetes mellitus for more than two years duration were included in our study. We excluded pregnant females, and patients suffering from any acute illness or any known psychiatric problem. Assuming the prevalence of poor sleep quality as 50% among diabetics and 15% as the permissible level of error, the sample size worked out to be 171. Considering the non-response rate of 20%, the sample size was fixed at 205 with the help of open Epi software. 

Data collection 

The eligible subjects who presented to the IDGDC were selected by modified systematic random sampling. After obtaining informed consent from the patients a face-to-face interview was done by trained physicians and public health workers to gather information using a pre-designed and pretested questionnaire [7-10]. The first section of the questionnaire was used to document the socio-demographic variables like age, sex, education, and work status over the past year along with the information related to diabetes mellitus such as disease duration, treatment (medications prescribed, dietary plan, etc.) and Glycosylated hemoglobin (HbA1C) levels. The last section of the proforma was used to gather information regarding the sleep quality of the patients, based on the Pittsburgh Sleep Quality Index (PSQI). The PSQI assesses sleep quality during the previous month. The PSQI consists of nineteen self-rated questions and five questions rated by the bed partner or roommate. The latter five questions are used for clinical information only and are not tabulated in the scoring of PSQI score. The 19 self-rated questions assess a wide variety of factors relating to sleep quality, including estimates of sleep duration and latency and of the frequency and severity of specific sleep-related problems. These I9 items are grouped into seven component scores, each weighted equally on a 0-3 scale. The seven component scores are then summed to yield a global PSQI score, which has a range of 0-21; higher scores indicate worse sleep quality [11-13].

Measures

Age, gender, area of residence, level of education, and current working status were assessed using a standardized questionnaire. Working status was categorized as employed and unemployed. Education was categorized as no formal education, less than ten years of schooling, and more than ten years of schooling. The body mass index (BMI) was calculated by dividing the weight in kilograms and height in meters square.

A subject was defined as having diabetes if the diagnosis had been made by a doctor if the subject was receiving anti-diabetes therapy or if two fasting blood samples showed glucose concentration according to the WHO definition of diabetes [4]. Diabetes control was ascertained by obtaining Glycosylated hemoglobin level(HbA1C) values. Study subjects with HBA1c levels less than 7gm/dl were considered to have good glycemic control and more than 7gm/dl was categorized as poor diabetes control [4]. Poor quality sleepers were defined as a PSQI score of five or more while good quality sleep was defined as a PSQI score of less than five [12,13]. The BMI was categorized as normal (18.5 to 22.99), overweight (23 to 24.99), and obese (25 and above) [14].

Data analysis 

The data thus generated was checked for completeness and analyzed using SPSS (IBM Corp. Released 2013. IBM SPSS Statistics for Windows, Version 22.0. Armonk, NY: IBM Corp). Descriptive statistics including frequency, percentage, mean and standard deviation were used to understand the sample distribution of sleep quality and diabetes control. A chi-square test was used to determine the factors associated with sleep.

## Results

A total of 192 subjects were included in the study. Most of them (124) were above 50 years and their mean age was 52.7±10.3 years. The men outnumbered females (17:15) and 53% of the subjects belonged to urban areas. Two-fifths of the subjects (39.6%) were employed and more than half of the study subjects (52.2%) had received more than 10 years of schooling (Table 1). Around one-third of the study subjects (32.3%) were diagnosed with diabetes for more than 10 years; 98 subjects had glycosylated Hb less than 7gm/dl while 94 had more than 7gm/dl. Half of the subjects (50%) were overweight while 20% of them were obese (Table 1). One hundred ten subjects (57.3%) had a PSQI score ≥5 i.e. they were poor sleepers while 82 (42.7%) had a PSQI score < 5 i.e. they were good sleepers. The mean PSQI score was 6.70±3.32 (Table 2).

**Table 1:** Socio-demographic, anthropometric, and clinical characteristics of the study participants, (n=192).

Socio demographic characteristics	N(%)
Age group (in years)	
20-35	12(6.3%)
36-50	56(29.1%)
51-65	124(64.5%)
Sex	
Male	102(53.1%)
Female	90 (46.9%)
Area of residence	
Rural	90(46.9)
Urban	102(53.1)
Working Status	
Employed	76(39.6)
Unemployed	116(60.4)
Education	
No formal education	2(1)
Less than 10 years of schooling	84(43.8)
More than 10 years of schooling	106(55.2)
Duration of Diabetes	
<10 years	130(67.7)
≥10 years	62(32.3)
HbA1C	
< 7%	98(51)
≥ 7%	94(49)
Treatment	
Oral Hypo-glycemics Agent	130 (67.7)
Insulin	10(5.2)
Both (Insulin+OHA)	52 (27.1)
Body Mass Index	
Normal (18.5-22.99)	56(29.2)
Overweight (23.0 – 24.99)	96(50)
Obese (25.00 and above)	40(20.8)
Total	192(100)

**Table 2:** Sleep characteristics of study subjects (n=192). Mean PSQI score - 6.70±3.32

Sleep characteristics	n(%)
PSQI score	
< 5	82(42.7)
≥ 5	110(57.3)
Duration of sleep	
>7 hours	82(42.7)
5-6 hours	16(8.3)
<5 hours	8(4.2)
Quality of sleep perceived by the subjects	
Very good	44(22.9)
Fairly good	116(60.4)
Fairly bad	24(12.5)
Very bad	8(4.2)
Intake of medication for sleep	
Not during past one month	160 (83.3)
Less than once a week	2(1.0)
Once or twice a week	6(3.1)
Three or more times a week	24(12.5)
Total	192(100)

Poor sleepers were maximum in the age group of 20-35 years and there existed a significant association between age and PSQI score (p-value 0.04). The number of poor sleepers were more in females though they were equally distributed among subjects of urban and rural area. 62% of the unemployed subjects were poor sleepers. There was no association between the education, residence, or employment status of the study subjects and their PSQI score (Table 3).

**Table 3:** Association between socio-demographic characteristics with sleep quality of study participants (n=192).

Socio-demographic Variable	PSQI<5 n(%)	PSQI≥5 n(%)	Total n(%)	p value#
Age Group(in years)				
20- 35	2(16.6)	10(83.4)	12	0.04 *
36-50	34(60.7)	22(39.3)	56	
51 and above	46(37.1)	78(62.9)	124	
Sex				
Female	34(37.8)	56(62.2)	90	0.354
Male	48(47.1)	54(52.1)	102	
Area of residence				
Rural	38(42)	52 (58)	90	> 0.05
Urban	44(43)	58(57)	102	
Working status				
Employed	38(50)	38 (50)	76	0.24
Unemployed	44(37.9)	72(62.1)	116	
Education level				
No formal education	2 (100)	0	2	0.487
Less than 10 years of schooling	34(40.4)	50(59.6)	84	
More than 10 years of schooling	46(43.4)	60(56.6)	106	
Total	82(42.7)	110(57.3)	192	
*statistically significant # Association estimated by Chi-square test				

There existed no significant association (p-value) between glycosylated Hemoglobin and PSQI score though 59% of the subjects with HBa1C more than 7gm/dl were poor sleepers. Among 110 poor sleepers, 32 subjects perceived that their sleep was very bad or fairly bad. Poor sleepers were more (74.2%) among study participants with a duration of diabetes of more than 10 years and among individuals with a BMI of more than 25 (75%), though it was found to be statistically insignificant (Table 4).

**Table 4:** Association between anthropometric and clinical characteristics with sleep quality of study participants (N=192).

Variable	PSQI<5 n(%)	PSQI≥5 n(%)	Total n	p value #
Duration of Diabetes				
Less than 10 years	66(50.7)	64 (49.3)	130	0.02*
More than 10 years	16(25.8)	46 (74.2)	62	
HbA1c				
< 7gm/dl	44(44.9)	54(55.1)	98	0.65
≥ 7gm/dl	38(40.4)	56(59.6)	94	
Treatment				
Insulin	4(40)	6(60)	10	0.915
OHAs	54(41.5)	76(58.5)	130	
Both	24(46.1)	28(53.9)	52	
BMI				
Normal (18.5-22.9)	26(42.3)	30(57.7)	56	0.16
Overweight (23-24.99)	46(48)	50(52)	96	
Obese(≥25.00)	10 (20)	30(75)	40	
Sleep quality perceived by Pts				
Very good	36(81.8)	8(18.2)	44	0.00*
Fairly good	46(39.6)	70(64.4)	116	
Fairly bad	2(8.3)	22(91.7)	24	
Very bad	1(12.5)	7(87.5)	8	
Total	82	110	192	
*statistically significant # Association estimated by Chi-square test				

## Discussion

The World Health Organization (WHO) has commented that diabetes is like an epidemic now. It is strongly associated with changes in the lifestyle of humans. It has been projected that DM will be the seventh leading cause of death [7]. There are a multitude of factors that affect the sleep quality in patients with type 2 diabetes mellitus. In turn, sleep quality has a great influence on the glycemic control of patients with DM. The present study was conducted in IDGDC of IQ City Medical College and NM Hospital, to assess the sleep quality in type 2 diabetes mellitus patients.

The result of the present study indicated that 57.3% of the subjects were classified as poor sleepers (PSQI> 5). The value obtained was higher compared to other studies conducted across the globe [8-9] which have reported 38 - 48 % 0f their subjects as “poor sleepers” but lesser as compared to a similar study conducted in India by Vigg et al. [10].

Our study reported a mean PSQI score of 6.70 ±3.32 which was more than that of a study conducted in Iran (5.5±4.4) [8] and lesser in comparison to the values reported by Vigg et al. (8.3±4.6) [10]. The mean duration of sleep per night as reported by the study subjects (6.3±0.95 hours ) was slightly more than (6.10±166 hours) as reported by Vigg et al. [10].

We obtained a significant association between age and PSQI score (Table 3) but no such significant association was seen in earlier studies [8-10]. An adult needs seven hours of sleep per night. The number of hours of sleep decreases with age. As more than 60% of the subjects in the present study were above 50 years of age, this finding may reflect their need for sleep during aging and the very change of intrinsic sleep processes at this phase of life.

Our study showed that subjects with more than 10 years duration of DM had a significant association with PSQI score (Table 4) which is similar to other studies [4,11]. Diabetic patients have an inability to maintain normal sleep patterns due to an imbalance between insulin secretion and glucose uptake [10].

Unlike our study, a significant relationship between HbA1c and PSQI score has been reported [10]. 75% of the obese subjects of our study were poor sleepers (Table 3) Vigg et al. [10] and Cunha et al. [11 ] also reported similar findings. Similar to our study no significant association was found between PSQI score and BMI in these studies too. Some studies have shown that obesity is correlated with insulin resistance in diabetes, and insulin resistance is significantly correlated with sleep quality [12].

In the present study, the percentage of poor sleepers was slightly higher, though statistically insignificant, among those treated through insulin injection alone or with OHAs (Table 4). Shamshigran et al. [8] also reported similar findings. Patients who are on insulin have a more severe form of the disease. They are more likely to have co-morbidities and a greater pressure of maintaining glycemic control which may further drive variations in glucose control and thus altered sleep quality.

In our study, we found that 160 patients rated their sleep quality as good. However, 50% of these subjects were poor sleepers and this association was statistically significant (Table 4). These patients may not bring sleep issues during their visit to health care providers hence, it is important to assess sleep in patients with DM with poor glycemic control in order to have a holistic view of the problem.

This study has some strengths and limitations. There are very few studies in India assessing the sleep quality in type 2 DM patients. Hence the results of this study can provide valuable information for clinicians to improve the management of diabetes. In our study, we included 192 subjects from a dedicated center for diabetes. The patients referred to this center might not be a representative sample of the entire population with diabetes. The information on sleep duration and sleep quality is self-reported and subjective. Thus, we cannot compare our results directly with other studies that used polysomnographic measures for sleep disturbance. We did not take into consideration the intake of caffeine, alcohol, other substance abuse, and breathing disorders which might have an effect on sleep quality and pattern. A large-scale multi-centric prospective study including diabetic patients from the general population comparing their sleep patterns with the glycemic index needs to be conducted. A subset analysis of the data thus generated based on various modifiable and non-modifiable risk factors for diabetes and poor sleep patterns will further clear the picture and firmly establish the association between them.

## Conclusions

In this study, a high proportion i.e. two-thirds of type 2 diabetes mellitus patients were found to have poor quality of sleep. The data supported an association between age and sleep quality. Subjects with prolonged duration of diabetes mellitus had poorer quality of sleep. Thus we can conclude that sleep quality is affected in patients with diabetes mellitus. The diabetes patient may not visit the healthcare provider with issues related to sleep. It is important on the part of the health personnel to attend the sleep issues and impaired quality of life due to sleep inadequacy. This will have an effect on the control of diabetes in addition to the quality of life of these patients. Hence sleep hygiene should be a part of diabetes management.
